# The trajectory of anxiety symptoms during the transition from childhood to young adulthood is predicted by IQ and sex, but not polygenic risk scores

**DOI:** 10.1002/jcv2.12268

**Published:** 2024-07-17

**Authors:** Ana Beatriz Ravagnani Salto, Giovanni A. Salum, Maurício Scopel Hoffmann, Marcos L. Santoro, André Zugman, Pedro M. Pan, Sintia I. Belangero, Lucas Toshio Ito, Victoria Fogaça Doretto, Marcos S. Croci, Marcelo J. A. A. Brañas, Carina de Giusti, Francisco Da Silva‐Jr, Sahâmia Martins Ribeiro, Euripedes Constantino Miguel, James F. Leckman

**Affiliations:** ^1^ Department & Institute of Psychiatry Faculdade de Medicina Universidade de São Paulo (USP) São Paulo São Paulo Brazil; ^2^ Department of Psychiatry Universidade Federal do Rio Grande do Sul (UFRGS) Porto Alegre RS Brazil; ^3^ Child Mind Institute New York New York USA; ^4^ Department of Neuropsychiatry Universidade Federal de Santa Maria (UFSM) Santa Maria RS Brazil; ^5^ Graduate Program in Psychiatry and Behavioral Sciences UFRGS Porto Alegre RS Brazil; ^6^ Departamento de Bioquímica da Universidade Federal de São Paulo (UNIFESP) Disciplina de Biologia Molecular São Paulo Brazil; ^7^ The Laboratory of Integrative Neuroscience (LINC) Universidade Federal de São Paulo (UNIFESP) São Paulo Brazil; ^8^ Department of Morphology and Genetics Universidade Federal de São Paulo (UNIFESP) São Paulo Brazil; ^9^ Yale Child Study Center Yale School of Medicine Yale University New Haven Connecticut USA

**Keywords:** adolescence, anxiety, cognition, intelligence, longitudinal

## Abstract

**Background:**

Understanding the factors that determine distinct courses of anxiety symptoms throughout development will better guide interventions. There are scarce data‐driven longitudinal studies, using multi‐modal predictors, investigating the chronicity of anxiety symptoms from childhood to young adulthood, particularly in a middle‐income country.

**Methods:**

2033 youths (ages 6–14 years [Mean age = 10.4 ± 1.94) at Baseline] were enrolled in the Brazilian High‐Risk Cohort for Mental Conditions longitudinal study, and assessed at three timepoints, between 2010 and 2019, using the Screen for Child Anxiety Related Disorders. Confirmatory Factor Analysis provided input to Growth Mixture Models to identify the best fitting trajectory model. Multinomial logistic regression analyses tested the effects of intelligence quotient (IQ), environmental factors and polygenic risk scores on internalizing symptomatology within trajectory class membership.

**Results:**

The best model solution identified three classes: high‐decreasing, moderate/low‐stable and low‐increasing symptoms over time. The high‐decreasing class showed a higher incidence of anxiety symptoms at the second time point (Mean age = 13.8 ± 1.93); while anxiety symptoms were highest in the low‐increasing class at the third timepoint (Mean age = 18.35 ± 2.03). Further, lower IQ predicted membership in the high‐decreasing trajectory class (OR = 0.68, 95% CI [0.55, 0.85]), while higher IQ predicted membership in the low‐increasing trajectory class (OR = 1.95, 95% CI [1.42, 2.67]). Finally, females were more likely than males to be in the low‐increasing trajectory class. Polygenic risk scores were not associated with anxiety trajectory class membership.

**Conclusion:**

Recognizing that anxiety symptoms follow diverse paths over time will allow for more effective intervention strategies. Specifically, interventions could accommodate children for greater anxiety risk in early childhood (i.e., lower IQ) versus late adolescence (i.e., higher IQ). That said, the emotional needs of girls in late adolescence should be monitored, regardless of their cognitive abilities or high achievements.

## Graphical abstract

Three distinct trajectories of anxiety symptoms were identified across three assessments spanning from childhood to young adulthood: a moderate/low stable class, a high‐decreasing class, and a low‐increasing class. Female sex and higher IQ predicted the low‐increasing trajectory, while a lower IQ predicted the high‐decreasing trajectory. These findings have significant implications for understanding how anxiety symptoms evolve over time and the contributing factors.

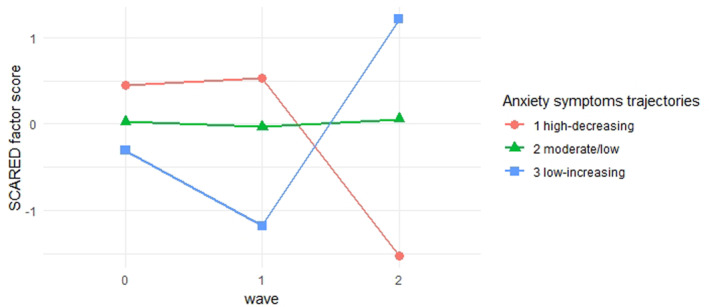

**Key points**

- There is a scarcity of data‐driven longitudinal studies investigating the chronicity of anxiety symptoms from childhood to young adulthood.
- Previous studies usually evaluate individual predictors, lacking inclusion of multimodal analyses.
- We identified three distinct trajectories of anxiety symptoms across three assessments spanning from childhood to young adulthood: a moderate/low stable class, high‐decreasing class, and low‐increasing class.
- Female sex and higher IQ predicted the low‐increasing trajectory, while lower IQ predicted the high‐decreasing trajectory. PRS were not associated with anxiety trajectories.
- These findings could impact clinical practice by tailoring interventions, as children with lower IQ are at risk for developing anxiety early in childhood while girls and children with higher IQ show an increase in symptoms in late adolescence.

## INTRODUCTION

Anxiety symptoms are extremely common during both typical and atypical development and exhibit variable trajectory courses over time (Beesdo et al., 2009; Penninx et al., 2021). Interestingly, the majority of people who experience anxiety symptoms in childhood and adolescence will develop to be adults with no diagnosable anxiety disorders (Beesdo et al., 2009). At the same time, other individuals will present with consistent patterns of diagnosable anxiety symptoms that will cause significant impairment and suffering into adulthood (Hovenkamp‐Hermelink et al., 2021; Penninx et al., 2021). Understanding the factors that determine the distinct trajectories of anxiety symptoms throughout development could help direct interventions to children who are at the highest risk for symptom chronicity and optimize resources to deliver treatment to those that need it most.

The definition of anxiety chronicity varies across studies (Hovenkamp‐Hermelink et al., 2021). One useful strategy is to define chronicity using data‐driven methods (Wardenaar et al., 2014), because these methods provide information that is not biased toward the pre‐existing clinical literature. That said, data‐driven methods may still differ in the way they define target groups. For example, some previously published studies have used subdomains of anxiety (Broeren et al., 2013; de la Torre‐Luque et al., 2020; Morales‐Muñoz et al., 2022), while others used multiple domains of symptoms (Allan et al., 2014; Crocetti et al., 2009; Duchesne et al., 2010; Prinzie et al., 2014; Spence et al., 2022). Few have used validated anxiety symptom scales, such as the Screen for Child Anxiety Related Disorders (SCARED) questionnaire (Crocetti et al., 2009).

Only three previous data‐driven studies have examined the trajectory of self‐reported anxiety symptoms during the transition from adolescence into early adulthood using validated anxiety scales. To elaborate, a four‐year study (1313 participants aged 10–20 years at baseline, using the SCARED scale [annually]) found two anxiety trajectory classes: high‐increasing (8.7%) and low‐decreasing (91.3%) symptoms (Crocetti et al., 2009). A two‐year study (277 participants aged 9–13 at baseline, using the Revised Child Anxiety and Depression Scales [annually]), found a three class solution: high‐increasing (10.5%), moderate‐decreasing (50.9%) and low‐decreasing (38.5%) symptoms (Allan et al., 2014). Finally, a four‐year study (3392 participants aged 12–13 years at baseline, using the Children's Anxiety Scale [at two‐year intervals]) also found three anxiety symptom classes: stable‐low (89.5% males; 78.2% females), low‐increasing (5.6% males; 14.4% females) and high‐decreasing (4.9% males; 7.4% females) symptoms (Spence et al., 2022). In addition, we note that a six‐year study using parent reports of their children (290 children aged 8–10 years at baseline, using the anxiety subscales of the Child Behavior Checklist [at 3‐year intervals]) found a three‐class solution: steadily low (82%), moderate increasing, then decreasing (5.9%) and high‐decreasing (12.1%) symptoms (Prinzie et al., 2014). Though there is variability, these studies are consistent in that all studies observed high levels of anxiety in about 10% of their samples, and three of four studies found that a three‐class solution best fit the data.

The predictors of anxiety chronicity have varied across studies. Most studies found that the course of anxiety symptoms was affected by a number of variables including the sex of the child, with girls demonstrating a greater risk for high symptom trajectories (Broeren et al., 2013; Crocetti et al., 2009; de la Torre‐Luque et al., 2020). Other variables include: maternal mental health disorders (Bushnell et al., 2020; Morales‐Muñoz et al., 2022), the level of childhood adversity (Duchesne et al., 2010), low levels of support from parents and teachers (Spence et al., 2022) and the level of anxiety sensitivity (Allan et al., 2014). There are no previous studies that used multimodal analyses to investigate the role of cognitive, genetic, environmental, and demographic factors in predicting these outcomes.

Intelligence has been previously associated with anxiety levels and again the results are mixed. For example, both high and low intelligence quotients (IQ) have been associated with anxiety (Kermarrec et al., 2020; Melby et al., 2020). Most studies have focused on specific populations such as children with autism spectrum disorders, intellectual disabilities, borderline IQ, or gifted children. Thus, there are few studies examining the association between intelligence and anxiety in community samples, and those that exist are cross‐sectional in nature and limited to categorical diagnoses (Keyes et al., 2017; Mahony et al., 2023).

Polygenic risk scores (PRS) are predictors of the genetic susceptibility to mental disorders (Murray et al., 2021). Polygenic risk scores are computed from genome wide association studies (GWAS) as a weighted sum of the number of risk alleles carried by an individual (Duncan et al., 2019). Despite being a useful tool to assess risk for psychiatric disorders and symptoms, PRS have not been evaluated in anxiety trajectories studies. A data‐driven longitudinal study can further elucidate how IQ and PRS affect anxiety beyond categorical diagnosis.

The current literature is limited in a few important ways. First, there are scarce data‐driven studies investigating the chronicity of anxiety symptoms from childhood to young adulthood. Second, existing studies focus on very specific domains of anxiety. Third, they are usually based on small samples. Fourth, the inclusion of multimodal predictors is rare. Fifth, the study population is typically recruited from high‐income countries. To address these gaps in the literature, here, we used a large Brazilian (i.e., middle‐income country) prospective longitudinal study of children and young adults to evaluate anxiety chronicity using multi‐modal predictors, namely, a data‐driven method, growth mixture model (GMM). With this approach, we evaluated the effects of cognitive, genetic, environmental, and demographic factors on anxiety trajectories.

The present study had three main aims. The first aim was to identify the trajectories of anxiety symptoms in youth aged from 6 to 22 years – measured at three datapoints between 2010 and 2019. The second aim was to examine the impact of intelligence, sex, threat exposure and maternal mental health disorders on the course of anxiety trajectories. The third aim was to determine if PRS for anxiety, depression and subjective wellbeing (SWB) could predict anxiety trajectory. We hypothesized that two to three classes of anxiety trajectories would best describe our sample, in line with previous studies (Allan et al., 2014; Crocetti et al., 2009; Spence et al., 2022). We also hypothesized that IQ, PRS, sex, threat exposure and maternal mental health disorders would predict anxiety trajectory class membership. Considering previous work on intelligence and anxiety, we hypothesized that both low and high IQ could be related to divergent anxiety trajectories (Kermarrec et al., 2020; Melby et al., 2020).

## MATERIALS AND METHODS

### Participants

Participants were children and adolescents enrolled in the Brazilian High‐Risk Cohort for Mental Conditions (BHRCS). This is a community‐based longitudinal study designed to the examine different aspects of child and adolescent mental health and development, using behavioral assessments, risk factors, neuropsychological examination, genetics, and neuroimaging. Details about the cohort can be found elsewhere (Salum et al., 2015). All parents signed informed consent and children provided verbal assent. The ethics committees of all three Brazilian universities involved in studying the cohort approved the project.

In 2010, 2511 children aged 6–14 years from 57 schools in two Brazilian cities, São Paulo and Porto Alegre, were enrolled in the study. From this pool, 958 children were randomly selected and 1553 were selected in a risk‐prioritization procedure that was conducted to identify individuals with current symptoms and/or a family history of psychopathology. The baseline assessment phase was performed across multiple visits and included a parent interview and 3 sessions of child evaluation. The parent interview consisted of a household lay interview with a biological parent (the mother in 94.5% of cases) and included a detailed evaluation of general risk factors for mental conditions and a structured psychiatric interview using the Developmental and Well‐Being Assessment (DAWBA), Brazilian Portuguese version (Fleitlich‐Bilyk & Goodman, 2004; Goodman et al., 2000). The child evaluation was administered by trained psychologists and speech therapists, and included the assessment of internalizing symptoms including anxiety, neuropsychological evaluation, and school performance.

Three waves of assessments were conducted where parents and children were interviewed and evaluated. Of the 2511 youth and parents who participated in the baseline assessment (years 2010‐11), 2010 youth and parents (80% of the original sample) participated again in the second assessment (years 2013–2015), and 1905 youth and parents (75%) participated in the third assessment (years 2017–2019). The final sample used in this study to compute anxiety trajectories consisted of 2033 youth who had at least two assessments of self‐reported anxiety symptoms using the SCARED (see “Measures” below). We included only youth with at least two assessments to adequately model long‐term continuity and change. As there were few missing cases in the cognitive evaluation, the sample for the multinomial logistic regression included 1912 youths. The sample for the analysis that included genetic data had 1758 youths because not all cases had a viable sample for genotyping.

### Measures

#### Anxiety assessment

The SCARED (Birmaher et al., 1997; Isolan et al., 2011) is a 41‐item questionnaire that was administered to evaluate anxiety symptoms in all three waves of data collection. Participants responded on a 3‐point Likert scale of 0 (Not True or Hardly Ever True), 1 (Somewhat or Sometimes True), or 2 (Very True or Often True). The scale works well, not only in clinical samples, but also in youth from the general Brazilian population (Isolan et al., 2011).

#### Sociodemographic variables

Sociodemographic data included age, sex, site of data collection, socioeconomic status, self‐declared skin color, and the presence of a maternal psychiatric disorder at baseline. A national official instrument was used to ascertain socioeconomic status (The Economic Classification Criterion Brazil) (ABEP, 2010). This instrument stratifies the population in five levels (A – wealthiest to E – poorest) and considers level of education of the head‐of‐household and purchase capacity. The two wealthiest and the two poorest categories were merged here, creating three groups (0 – low, 1 – middle and 2 – high socioeconomic status). Skin color was assessed by self‐report and categorized as either white or non‐white. The presence of a maternal psychiatric disorder was assessed at baseline using the Mini International Psychiatric Interview (MINI) and the MINI Plus (Amorim et al., 1998; Sheehan et al., 1998).

#### Threat exposure

A factor score to measure threat exposure was computed using factor analysis as previously reported (Schafer et al., 2023). The variables included in the analysis were drawn from the Posttraumatic Stress Disorder assessment of the DAWBA (Goodman et al., 2000) and questionnaires specifically designed for the BHRCS (Salum et al., 2015). Further details on the computation of the threat exposure factor score can be found in the article (Schafer et al., 2023).

#### Intelligence quotient

Intelligence quotient (IQ) was estimated using the vocabulary and block design subtests of the Wechsler Intelligence Scale for Children, third edition (Wechsler, 2002), using the Tellegen and Briggs method (Tellegen & Briggs, 1967) and Brazilian norms (Figueiredo, 2001). IQ was standardized using *z* scores.

#### Polygenic risk scores

Polygenic risk scores were calculated for depression, anxiety disorders and SWB. The 2033 youths included in the trajectory analysis were genotyped. After conducting a quality control assessment, 1758 participants were selected and included in the analysis.

Genomic DNA was isolated from saliva (Oragene) using prepIT‐L2P reagent (DNAgenotek). Genotyping was performed using the Global Screening Array (Illumina). Single‐nucleotide variants with a minor allele frequency <1%, locus missingness >10%, or Hardy‐Weinberg equilibrium significance <0.000001 were excluded, as were individuals with genotype missingness >10% and an estimation of identity by descent >0.12.

Polygenic risk scores were calculated with the PRS‐CS software (Ge et al., 2019), which applies a continuous Bayesian shrinkage parameter to infer the mean posterior effect size for each Single‐nucleotide variants, based on GWAS summary statistics using a European Linkage Disequilibrium (LD) panel from 1000 Genomes Project. The PRS‐CS global shrinkage parameter was learned from the data using a fully Bayesian approach and other PRS‐CS parameters were set as default. All SNVs that overlapped within the target sample, discovery GWAS summary statistics and the LD panel were included in the analysis of the PRS. The Plink v1.9 score function was used to calculate individual PRS, which were standardized afterward in R to a mean = 0 and SD = 1.

The PRS for depression were computed based on the GWAS meta‐analysis of 294,322 cases and 741,438 controls (Als et al., 2023). The PRS for anxiety were computed based on the GWAS meta‐analysis of 5761 cases and 11,765 controls (Otowa et al., 2016). The PRS for SWB PRS were computed based on the GWAS of 298,420 individuals (Okbay et al., 2016). The 10 first principal components were included as covariates in all the models that included the PRS.

### Data analysis

#### Confirmatory Factor Analysis

A measurement invariance analysis was performed to evaluate if the latent construct of the items of the SCARED questionnaire was valid across multiple timepoints. For this, the package “lavaan” (Rosseel, 2012) and the package “SemTools” (Rosseel et al., 2022) were used in R [Version 4.1.2] (R Core Team (2021)) and RStudio [Version 2021.9.0.351] (RStudio Team (2021)). We tested configural, metric, scalar, and strict invariance by considering the comparative fit index (CFI), the standardized root mean square residual (SRMR), and the root mean square error of approximation (RMSEA). Measurement invariance was supported when ∆CFI > −0.01 or ∆SRMR <0.015, or ∆RMSEA <0.030 (for metric invariance) or 0.015 (for scalar or residual invariance) (Chen, 2007).

As sum‐scores assume that all items inform the construct with the same importance (i.e., equal factor loadings), we derived factor scores based on Confirmatory Factor Analysis (CFA), which reflect values of latent measures for each individual (Distefano et al., 2008).

Participants from the BHRCS that had at least two assessments of self‐reported anxiety data measured with the SCARED questionnaire were included in the analysis, totaling 2033 individuals. We excluded participants with only one assessment to adequately model long‐term continuity and change, consistent with previous studies (Ahlen & Ghaderi, 2020). Participants with only one SCARED measurement were more likely to report white skin color (*χ* ^2^ = 9.6, *p* = 0.0019, *V* = 0.06) and belong to a low economic status group (*χ* ^2^ = 6.5, *p* = 0.039, *V* = 0.05). No significant differences were observed in terms of site, sex, maternal mental disorder, threat exposure, age, and IQ. Confirmatory Factor Analysis was performed using the Mplus software (version 8.6). The data were set in long format; timepoints were nested within individuals which were then considered as clusters. After that, we calculated and extracted factor scores for the one factor model using standard Mplus procedure. Confirmatory Factor Analysis was carried out using delta parameterization and weighted least squares with diagonal weight matrix with standard errors and mean‐ and variance‐adjusted chi‐square test statistics (WLSMV) estimators. Full Information Maximum Likelihood approaches were used to allow retention of individuals with missing data. To evaluate model fit, we used RMSEA, CFI and Tucker–Lewis index (TLI). Values of RMSEA lower than 0.060 and CFI or TLI values higher than 0.950 indicate a good‐to‐excellent model (Hu & Bentler, 1999).

From the total 2033 participants with at least two measurements of SCARED, there were 1256 complete cases and 777 with one datapoint missing. We examined differences between participants with two and three SCARED measurements. While a statistically significant difference in IQ was observed (*p*‐value = 0.019), the clinical relevance was minimal, with a mean IQ difference of only two points (mean IQ = 102.5 for the three SCARED measurements group and mean IQ = 100.6 for the two SCARED measurements group). No significant differences were found in other variables, including sex, age, site, skin color, maternal mental disorder, and threat exposure. We imputed the missing values of the SCARED factor scores with the nearest value (i.e. missing first and third measurements imputed with second measurement). If the second measurement was missing, it was imputed with the mean value of the first and third measurements.

Finally, age and sex were regressed from the SCARED factor scores in a generalized additive model and residuals were saved for the Growth Mixture Modeling (GMM) analysis. For this, the package “mgcv” (Wood, 2011) was used in R [Version 4.1.2] (R Core Team (2021)) and RStudio [Version 2021.9.0.351] (RStudio Team (2021)) (Figure S1).

#### Growth Mixture Modeling

Anxiety trajectories were estimated with GMM (Muthen & Muthen, 2000) based on SCARED factor scores (see above). Growth Mixture Modeling has been widely used to study anxiety trajectories (Allan et al., 2014; Broeren et al., 2013; Crocetti et al., 2009; Prinzie et al., 2014). It can identify the smallest number of trajectory classes that capture the most variance among individuals. Growth Mixture Modeling allows random variability around the mean trajectories within each trajectory class. We preferred to use GMM because group‐based trajectory modeling, another method used to compute trajectories, assumes that repeated measures of the same individual are independent (Proust‐Lima et al., 2017), which can interfere in the proper classification.

To determine the model that best fit the data, mixture models with varying numbers of classes (one to six classes) were compared. For the comparison, the Bayesian Information Criterion (BIC), Log‐Likelihood (loglik), Size‐adjusted Bayesian Information Criterion (SABIC) and Akaike Information Criterion (AIC) were used. Furthermore, we evaluated the content and theoretical meaningfulness of the classes in the various solutions. The best fitting model determined the classes to be used for further analyses. Growth Mixture Modeling was carried out using the “lcmm” package (Proust‐Lima et al., 2017) in R [Version 4.1.2] (R Core Team (2021)) and RStudio [Version 2021.9.0.351] (RStudio Team (2021)).

#### Multinomial logistic regression

Multinomial logistic regression analyses were performed to test the effects of baseline cognitive, environmental, demographic, and genetic variables on trajectory class membership. All predictors were simultaneously entered into the models. Additionally, socioeconomic status, age, skin color and site were included as covariates to control for potential confounding factors and enhance the precision of the results. These covariates were selected based on their known associations with both the predictors and the anxiety trajectory classes. For example, variations in the site of data collection could introduce differences in threat exposure and influence class membership (Pei et al., 2022). Socioeconomic status and skin color may also contribute to variances in baseline maternal mental health disorders (Chen et al., 2019). Furthermore, age serves as a potential confounder for anxiety trajectory classes, baseline maternal mental disorders, and threat exposure (Gallo et al., 2017). By including these covariates, we aimed to ensure a more accurate assessment of the relationships between the predictors and anxiety trajectory classes.

For the multinomial logistic regression that included cognitive, environmental and demographic variables, we accounted for uncertainty of class membership using the “lcmm” package in R. For the model incorporating genetic variables, the “VGAM” package (Yee, 2015) in R was used, as the model with uncertainty of class membership did not converge. Failure to consider the uncertainty of class membership could elevate the risk of bias. However, we deemed this risk to be low, attributing it to the evident separation of anxiety symptom trajectories (Proust‐Lima et al., 2023) (refer to “Results” for entropy values).

## RESULTS

### Demographics and sample characteristics

Demographics and sample features of the 2033 participants are reported in Table 1. Male sex constituted 54.8% of the sample in the first wave. Mean age was 10.4 (SD = 1.94), 13.8 (SD = 1.93) and 18.35 (SD = 2.03) years, respectively in the first, second and third waves.

**TABLE 1:** Baseline demographics and total sample characteristics.

Variable	Frequency (percentage) or mean (SD)
Sex, n (%)	
Male	1115 (54.8)
Female	918 (45.2)
Skin color, n (%)	
White	1110 (54.6)
Non‐white	923 (45.4)
Site, n (%)	
São Paulo	1012 (49.8)
Porto Alegre	1021 (50.2)
Socioeconomic status, n (%)	
Low	223 (11)
Middle	1454 (71.5)
High	356 (17.5)
Any mental disorder^a^, n (%)	
Yes	544 (26.8)
No	1489 (73.2)
Current mother mental disorder^b^, n (%)	
Yes	612 (30.1)
No	1421 (69.9)
SCARED, mean (SD)	24.7 (14.5)
Age, mean (SD)	10.4 (1.94)
Threat factor loading, mean (SD)	0.07 (0.73)
IQ, mean (SD)	101.8 (16.9)

^a^ Based on the Development and Well‐Being Assessment (DAWBA) questionnaire.

^b^ Based on Mini International Psychiatric Interview. SD, standard deviation; IQ, intelligence coefficient.

### Confirmatory Factor Analysis

The unidimensional structure of the SCARED questionnaire was shown to be invariant across the assessments. Specifically, metric measurement invariance was supported by the fit indices of ∆CFI (0), ∆SRMR (0.019), and ∆RMSEA (−0.001). Additionally, scalar and strict invariance were supported by fit indices (scalar invariance: ∆CFI = 0, ∆SRMR = 0, and ∆RMSEA = 0.002; strict: ∆CFI = 0, ∆SRMR = 0, and ∆RMSEA = 0) (Table S1).

The one‐factor model analyzed for the CFA had good fit statistics (RMSEA = 0.015, CFI = 0.97, TLI = 0.969). The Omega coefficient for the one‐factor model was *ω* = 0.91, indicating high internal consistency. The calculated factor scores for the one‐factor model resulted in individual values for each datapoint (Figure S2 for individual factor score trajectory). The factor loading for each item of the SCARED can be found in Supplemental Table S2. The resulting individual SCARED factor scores were used in further analyses.

### Anxiety trajectories

**FIGURE 1:**
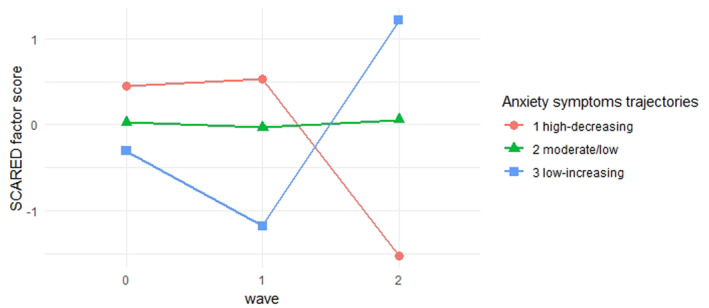
Anxiety symptoms trajectories from the best fitting three‐class growth mixture model (GMM). The *y*‐axis indicates the expected Screen for Child Anxiety Related Disorders (SCARED) factor score for a given class at a given timepoint. *SCARED, Screen for Child Anxiety Related Disorders*.

To identify classes with different anxiety trajectories we modeled latent classes of anxiety using GMM. The best fitting model was a three‐class model (presented in Figure 1).

**FIGURE 2:**
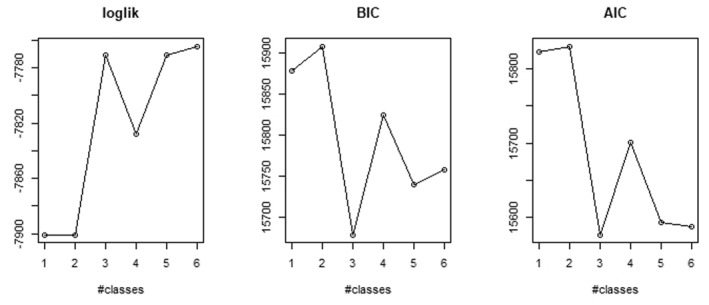
Fit indices across growth mixture models (GMMs) with different numbers of classes. The *x*‐axes represent models with 1‐6 classes, and the *y*‐axes show: Log‐likelihood (loglik), Bayesian Information Criterion (BIC), and Akaike Information Criterion (AIC) values.

Fit statistics for the GMMs with one, two, three, four, five and six classes were compared (presented in Figure 2 and Table 2).

**TABLE 2:** Fit indices, entropy for estimated growth mixture models (GMMs).

GMM	loglik	AIC	BIC	SABIC	Entropy
1 class	−7900.79	15,821.58	15,877.75	15,845.98	1
2 classes	−7900.79	15,829.58	15,908.22	15,863.74	0
**3 classes**	**−7770.56**	**15,577.13**	**15,678.24**	**15,621.05**	**0.89**
4 classes	−7828.07	15,700.13	15,823.71	15,753.82	0.82
5 classes	−7770.56	15,593.13	15,739.18	15,656.57	0.33
6 classes	−7764.34	15,588.69	15,757.21	15,661.89	0.44

*Note*: The best fitting model and its fit statistics are highlighted in bold.

Abbreviations: AIC, Akaike Information Criterion; BIC, Bayesian Information Criterion; GMM, Growth Mixture Modeling; Loglik, Log‐Likelihood; SABIC, Sample‐Size Adjusted Bayesian Information Criterion.

The three‐class solution was chosen because it had the lowest BIC, SABIC and AIC. Also, the three‐class model represented an inflection point in the BIC and AIC elbow plot (Figure 2). Classification accuracy of the three‐class model was good with an entropy value of 0.89. Also, the chosen model showed epidemiological coherence with most participants in the moderate/low‐stable trajectory class, a subgroup in a high‐decreasing trajectory class and a small group in the low‐increasing trajectory class.

The three anxiety symptom trajectories are presented in Figure 1 (their intercepts and slopes can be found in the Table S3). The first class (high‐decreasing) comprised 154 (7.58%) youth. It was characterized by a high initial level of anxiety that decreased over time. The second class (moderate/low‐stable) comprised the largest group with 1821 (89.57%) of the sample. It was characterized by moderate/low levels of anxiety that remained stable over time. The third class (low‐increasing) comprised 58 (2.85%) of the sample. This class had initially low levels of anxiety that increased over time.

There was a considerable age range at baseline, but most of the sample (approximately 70%) fell within the ages of 8–12 years at baseline, with a mean age of 10.4 (SD = 1.94). To assess whether differences in age could impact anxiety trajectories, we performed a Growth Mixture Analysis, dividing the sample into two groups: those below 10 years old (*n* = 1072) and those above 10 years old (*n* = 961). Importantly, we found similar anxiety trajectories in both subgroups as when analyzing the entire sample (Figure S3). Consequently, we opted to retain the entire sample in our analyses.

### Predicting class membership

First, correlations among the predictive variables were conducted to examine collinearity. The magnitude of correlations was low (−0.04 to −0.17), suggesting that the variables were relatively independent of each other. Next, multinomial logistic regression that accounted for uncertainty of class membership was used to predict class membership, setting the moderate/low stable class as the reference group against which the other two were compared.

Table 3 shows a summary of the model estimates and odds ratios for the predictors, including sex, baseline maternal mental disorder, threat factor loading and intelligence quotient (IQ), as well as the covariates socioeconomic status, age, site, and skin color. The total number of participants in this model was 1912 youths that had complete information for all the predictors.

**TABLE 3:** Summary of associations between predictors and anxiety symptoms trajectories.

Predictors	Moderate/low stable class (ref)	High‐decreasing class			Low‐increasing class		
		OR	95% CI	*p* value	OR	95% CI	*p* value
Sex							
Male	(Ref)	1.00			1.00		
Female		1.08	0.72–1.62	0.696	**2.72**	**1.36**–**5.43**	**0.004**
Skin color							
White	(Ref)	1.00			1.00		
Non‐white		1.09	0.73–1.63	0.679	0.82	0.43–1.56	0.543
Maternal mental disorder							
No	(Ref)	1.00			1.00		
Yes		1.16	0.75–1.79	0.516	1.12	0.54–2.32	0.771
Socioeconomic status							
Low	(Ref)	1.00			1.00		
Middle		0.95	0.54–1.68	0.867	1.35	0.60–3.04	0.473
High		0.75	0.34–1.66	0.482	1.00	0.62–1.61	0.989
Site							
São Paulo	(Ref)	1.00			1.00		
Porto Alegre		**1.94**	**1.25**–**3.00**	**0.003**	1.25	0.65–2.38	0.503
Age		0.91	0.82–1.01	0.075	0.93	0.79–1.10	0.394
Threat		1.19	0.91–1.55	0.207	0.67	0.40–1.10	0.116
Intelligence quotient		**0.68**	**0.55**–**0.85**	**0.001**	**1.95**	**1.42**–**2.67**	**<0.001**

*Note:* Significant predictors (*p* < 0.05) are printed in bold. IQ is represented in *z* score. The low‐increasing and high‐decreasing trajectories are compared with the moderate/low stable trajectory. Ref, reference category.

Girls had significantly greater odds than boys of being in the low‐increasing trajectory (OR = 2.72, 95% CI = 1.36–5.43, *p* = 0.004) in comparison to the moderate/low trajectory. Therefore, girls had higher risk of belonging to a trajectory of emerging anxiety symptoms in late adolescence. IQ was a significant predictor of membership in anxiety trajectory classes. More specifically, IQ was positively associated with the low‐increasing trajectory (OR = 1.95, 95% CI = 1.42–2.67, *p* < 0.001) and negatively associated with the high‐decreasing trajectory (OR = 0.68, 95% CI = 0.55–0.85, *p* = 0.001). Finally, children from the city of Porto Alegre had higher odds to be in the high‐decreasing trajectory (OR = 1.94, 95% CI = 1.25–3.00, *p* = 0.003). We also investigated whether PRS for anxiety, depression and SWB could predict class membership, but no relationship was found (Table 4). For this model, the total number was 1758 youths who had genotyping data.

**TABLE 4:** Summary of associations between predictors (including polygenic risk scores (PRS) and 10 first principal components) and anxiety symptoms trajectories.

Predictors	Moderate/low stable class (ref)	High‐decreasing class			Low‐increasing class		
		OR	95% CI	*p* value	OR	95% CI	*p* value
**Sex**							
Male	(Ref)	1.00			1.00		
Female		1.02	0.71–1.48	0.903	**2.07**	**1.16**–**3.70**	**0.014**
**Skin color**							
White	(Ref)	1.00			1.00		
Non‐white		0.913	0.56–1.49	0.716	1.36	0.63–2.96	0.432
**Maternal mental disorder**							
No	(Ref)	1.00			1.00		
Yes		1.15	0.76–1.72	0.513	0.960	0.49–1.88	0.906
**Socioeconomic status**							
Low	(Ref)	1.00			1.00		
Middle		0.839	0.5–1.41	0.510	2.43	0.57–10.32	0.228
High		0.626	0.3–1.31	0.212	2.64	0.56–12.33	0.218
**Site**							
São Paulo	(Ref)	1.00			1.00		
Porto Alegre		**1.66**	**1.1**–**2.51**	**0.016**	0.850	0.44–1.66	0.632
**Age**		**0.902**	**0.82**–**0.99**	**0.034**	0.951	0.82–1.1	0.507
**Threat**		1.14	0.88–1.48	0.307	0.852	0.55–1.32	0.475
**Intelligence quotient**		**0.728**	**0.6**–**0.89**	**0.002**	**1.51**	**1.12**–**2.03**	**0.006**
**PRS anx**		1.16	0.96–1.40	0.123	1.00	0.75–1.34	0.985
**PRS dep**		0.924	0.76–1.13	0.438	1.02	0.75–1.39	0.890
**PRS SWB**		1.03	0.85–1.24	0.780	1.04	0.78–1.39	0.780

*Note:* Significant predictors (*p* < 0.05) are printed in bold. IQ is represented in *z* score. The low‐increasing and high‐decreasing trajectories are compared with the moderate/low stable trajectory. First 10 principal components included in the model. PRS anx, polygenic risk score for anxiety disorders; PRS dep, polygenic risk score for depression; PRS SWB, polygenic risk score for subjective wellbeing; Ref, reference category.

## DISCUSSION

The present study aimed to evaluate and identify the progression of anxiety symptoms in children and young adults. Three distinct anxiety symptom trajectories were identified based on self‐reported information collected from youths aged 6–22 years over three timepoints (i.e., Mean ages = ∼ 10; ∼14; and ∼18; SDs ∼2 at all three timepoints). We found that IQ and gender, but not PRS, predicted anxiety trajectory class membership.

### Anxiety symptom trajectories

In line with previous studies of anxiety trajectories during adolescence, we found a three‐class solution for our sample (Allan et al., 2014; Prinzie et al., 2014; Spence et al., 2022). In addition, we observed that a class with low stable symptoms over time included most participants. As anxiety symptoms occur as part of normal development (Barzilay et al., 2020) and are present in the population in subclinical levels, it is possible that this trajectory represents the normal amount of anxiety present in the general population.

The other two classes we found – high‐decreasing and low‐increasing – are consistent with the findings of Spence et al. (2022) who also found similar trajectories. Our findings indicate that the high‐decreasing class is related to anxiety disorders prevalent during childhood such as separation anxiety and specific phobia and the low‐increasing trajectory is linked to disorders that manifest later in life including panic disorder/agoraphobia, social phobia and generalized anxiety disorder.

### Anxiety trajectories and IQ

We found that intelligence quotient (IQ) was related to the two anxiety trajectories that diverged from the moderate/low stable symptoms trajectory. Lower IQ predicted membership in the high‐decreasing trajectory, while higher IQ predicted membership in the low‐increasing trajectory. Thus, individuals who are at the extremes of the IQ spectrum might be at risk for developing anxiety symptoms at different ages: lower IQ being associated with high anxiety during childhood, while higher IQ is associated with high anxiety during adolescence/early adulthood. One previous study found increased anxiety disorders diagnosed by child psychiatrists in youth aged from 6 to 16 years with high intellectual potential (IQ > 130) compared to those with IQ below 130 (Kermarrec et al., 2020). However, it is not clear whether giftedness is associated with socio‐emotional and/or behavioral problems (Tasca et al., 2022). Studies on borderline or low IQ individuals found that these subgroups had higher rates of anxiety and other mental health diagnoses (Emerson, 2003; Melby et al., 2020).

Most previous studies in community samples report an association between lower IQ and increased mental health problems (Mahony et al., 2023). However, for internalizing disorders such as anxiety and depression this association is not consistent (Keyes et al., 2017). One study found that social and separation anxiety reported by parents and panic symptoms reported by youth were associated with lower IQ (Mahony et al., 2023). Conversely, measurements of general anxiety, other anxiety domains and depression were not related to IQ. Keyes et al. (2017) found that both specific phobia and separation anxiety were associated with lower IQ, while past‐year depression was associated with higher IQ. As specific phobia and separation anxiety are more prevalent during childhood, it is possible that anxiety symptoms within this age period are related to lower IQ, as we found in our high‐decreasing class showing a remission in symptoms in late adolescence/early adulthood. That said, Keyes et al. (2017) did not find that anxiety was related to higher IQ, as we found in the low increasing anxiety trajectory class.

One possible explanation for the association between lower intelligence and a predisposition to anxiety lies in the decline of the cognitive reserve capacity (Melby et al., 2020). It has been suggested that cognitive reserve serves as a protective factor for psychopathology. A diminished cognitive reserve may result in less effective coping strategies, thereby increasing vulnerability to anxiety. It is important to note that, in the present study, we observed this association specifically during late childhood/adolescence (i.e., at the first and second assessments, average age ∼10 ± 2 and ∼14 ± 2, respectively).

Conversely, higher IQ could predispose individuals to anxiety due to cognitive maturity, leading to anticipatory anxiety related to complex concepts or social preoccupations that can be challenging at a young age (Kermarrec et al., 2020). Additionally, it has been proposed that perfectionism could play a role in internalizing problems among gifted youth, as negative emotions may stem from high standards that prove to be unattainable. However, this hypothesis requires further investigation (Guignard et al., 2012). These findings may be consistent with our present finding of high IQ being related to anxiety symptoms in late adolescence (i.e., third assessment, average age ∼ 18 ± 2).

### Anxiety trajectories, sex, and polygenic risk scores

We found that girls were more likely to follow the low‐increasing trajectory. Previous anxiety trajectory studies have found that girls were more likely to be in high or increasing anxiety classes (Allan et al., 2014; Crocetti et al., 2009; de la Torre‐Luque et al., 2020; Spence et al., 2022), while others found no sex differences (Duchesne et al., 2010; Prinzie et al., 2014). These discrepancies could be explained by the fact that sex differences in anxiety symptoms may emerge after puberty, as the gender gap in the prevalence of anxiety disorders increases after the age of 13 (Bittner et al., 2007; Steinsbekk et al., 2022), with higher anxiety rates persisting in girls into adulthood (McLean et al., 2011). Thus, the higher prevalence of girls in the low‐increasing trajectory in our study may be related to this increase in anxiety symptoms in late adolescence and early adulthood.

Polygenic risk scores for anxiety, depression and SWB were not associated with any of the anxiety trajectories. One possible explanation for this negative finding is that our sample is ethnically diverse, as the Brazilian population has a multi‐ethnic and admixed background, while PRS are mostly based on European ancestry. It has been reported that the performance of PRS in non‐European populations is generally poorer, particularly for African ancestry samples (Duncan et al., 2019). Even though 10 principal components were included in the present study, the training and target samples used to build PRS were from different populations (Talarico et al., 2019). Another possibility is that the phenotypes studied in the GWAS used to build the PRS may have diverged from our anxiety trajectories; our trajectories were built from dimensional anxiety scores while the GWAS studies included participants with diagnosed anxiety, depression, or measures of SWB.

We expected to find that a positive history of maternal mental disorder would predict trajectory class membership as observed in previous reports (Bushnell et al., 2020; Duchesne et al., 2010; Morales‐Muñoz et al., 2022), but no association was found. We note that these previous reports of higher anxiety trajectories in youth were associated with specific maternal diagnoses within their family history, including postnatal maternal anxiety (Morales‐Muñoz et al., 2022) and depression (Bushnell et al., 2020; Duchesne et al., 2010; Morales‐Muñoz et al., 2022). In the present study, we included any maternal mental disorder assessed at baseline which could have affected our ability to detect an association with youth anxiety trajectories. Nevertheless, the impact of maternal mental health on youth anxiety requires further investigation.

Childhood adversity is a well‐established risk factor associated with the emergence mental health problems at all life‐course stages (Kessler et al., 2010). Here, we focused on a specific dimension of childhood adversity, namely threat exposure, and found no association with anxiety symptom trajectory. It is possible that different dimensions of adversity may have unique cognitive and socioemotional consequences that will require further investigation (Wade et al., 2022).

### Limitations

The present results should be considered in light of the following limitations. First, only self‐reported data were included in this study. A multiple‐informant approach could improve the reliability of information regarding the development of anxiety symptoms in youth. Another limitation was the inclusion of only three time points in determining trajectories, which could have undermined the discovery of other, more complex trajectories. Furthermore, despite being a school‐based sample, our sample may not have been representative of the entire Brazilian population as children were selected from only two Brazilian cities and a portion of the sample was oversampled for mental health risk factors. Additionally, the presence of missing data values could potentially introduce bias or reduce the statistical power of the analysis. Although techniques such as imputation were used to handle missing data, it is important to acknowledge that imputed values are estimates and may not accurately reflect true values. As such, the interpretation of results should be cautious, recognizing the potential impact of missing data on the validity and generalizability of findings.

## CONCLUSION

The findings from this study extend previous work on the course of anxiety symptoms by simultaneously investigating the impact of intelligence, sex, and genetics on anxiety trajectories. To summarize, the present study identified three distinct trajectories of anxiety symptoms across three assessments spanning from childhood to young adulthood: a moderate/low stable class, high‐decreasing class, and low‐increasing class. Further, female sex and higher IQ predicted the low‐increasing trajectory, while lower IQ predicted the high‐decreasing trajectory.

These findings have significant implications for understanding how anxiety symptoms evolve over time. Recognizing that anxiety symptoms follow diverse paths during this critical period allows for more tailored and effective intervention strategies. Mental health professionals should be attuned to the unique needs of individuals with anxiety; for example, adapting interventions to address cognitive factors in children experiencing anxiety. Additionally, it is important to monitor the emotional needs of girls in late adolescence, regardless of their cognitive abilities or high achievements.

## AUTHOR CONTRIBUTIONS

**Ana Beatriz Ravagnani Salto**: Conceptualization; Formal analysis; Investigation; Methodology; Project administration; Software; Validation; Visualization; Writing – original draft. **Giovanni Abrahao Salum**: Conceptualization; Funding acquisition; Methodology; Project administration; Resources; Supervision; Writing – original draft; Writing – review & editing. **Mauricio Hoffmann**: Data curation; Formal analysis; Investigation; Methodology; Software; Supervision; Validation; Visualization; Writing – original draft; Writing – review & editing. **Marcos Santoro**: Data curation; Formal analysis; Investigation; Methodology; Project administration; Software; Supervision; Writing – review & editing. **André Zugman**: Conceptualization; Methodology; Supervision. **Pedro Mario Pan**: Conceptualization; Funding acquisition; Investigation; Methodology; Project administration; Resources; Supervision. **Sintia Belangero**: Funding acquisition; Methodology; Resources; Software; Supervision; Writing – review & editing. **Lucas Toshio Ito**: Data curation; Formal analysis; Software; Validation; Visualization; Writing – original draft. **Euripedes Miguel**: Conceptualization; Funding acquisition; Project administration; Resources; Supervision; Writing – review & editing. **James F. Leckman**: Conceptualization; Funding acquisition; Methodology; Project administration; Resources; Supervision; Writing – review & editing.

## CONFLICT OF INTEREST STATEMENT

The authors have declared no competing or potential conflicts of interest.

## ETHICS STATEMENT

This study was approved by the ethics research committee of the Universidade de São Paulo. The BHRCS obtained approval from the ethics committees of all three Brazilian Universities involved in the cohort: Universidade de São Paulo, Universidade Federal de São Paulo, and Universidade Federal do Rio Grande do Sul.

## Supporting information

Supplementary Material

## Data Availability

The data that support the findings of this study are available on request from the corresponding author. The data are not publicly available due to privacy or ethical restrictions.
