# Supplementary material for: The trajectory of anxiety symptoms during the transition from childhood to young adulthood is predicted by IQ and sex, but not polygenic risk scores
Source: JCPP Adv. 2024 Jul 17;5(1):e12268. doi: 10.1002/jcv2.12268 (PMC11889643; doi:10.1002/jcv2.12268)
Supplement: Supplementary file 1 — Supplementary Material [file JCV2-5-e12268-s001.docx]

**The Trajectory of Anxiety Symptoms during the Transition from Childhood to Young Adulthood is Predicted by IQ and sex, but not Polygenic Risk Scores**

**Supporting information**


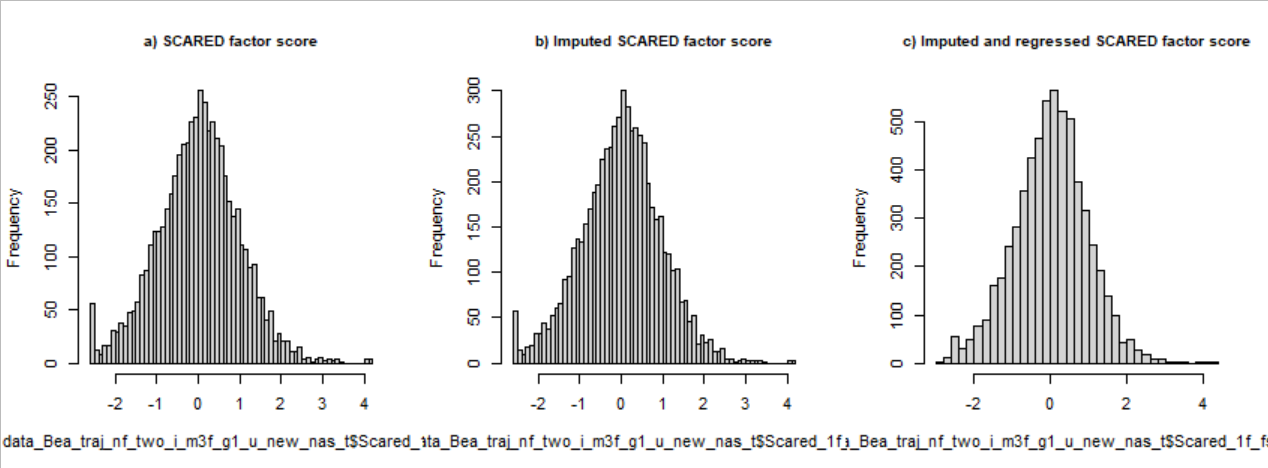


**Figure S1. Distribution of a) SCARED factor score, b) Imputed SCARED factor score and c) Imputed and regressed SCARED factor score**


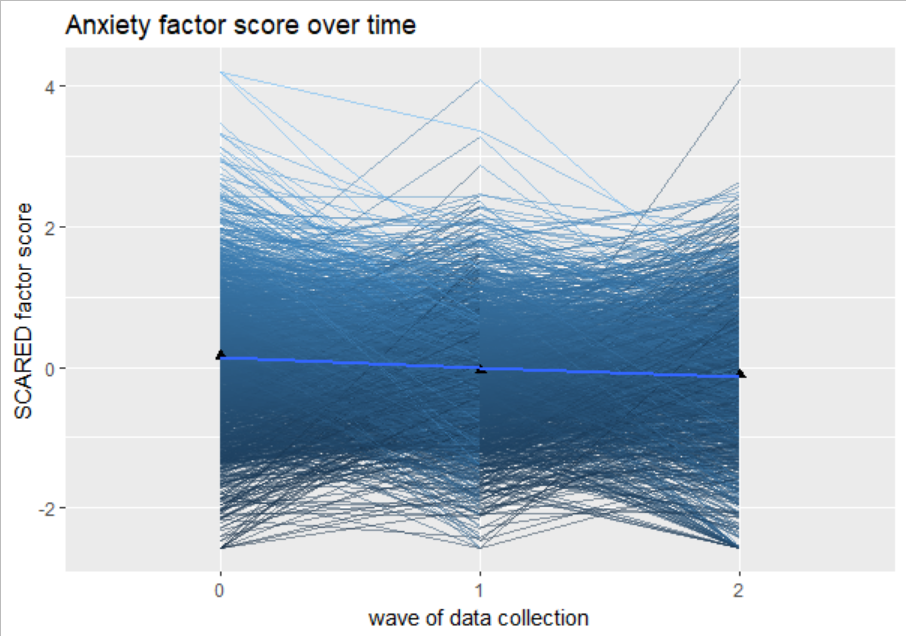


**Figure S2. SCARED factor score over time. Each line represents an individual (n=2,033)**

**
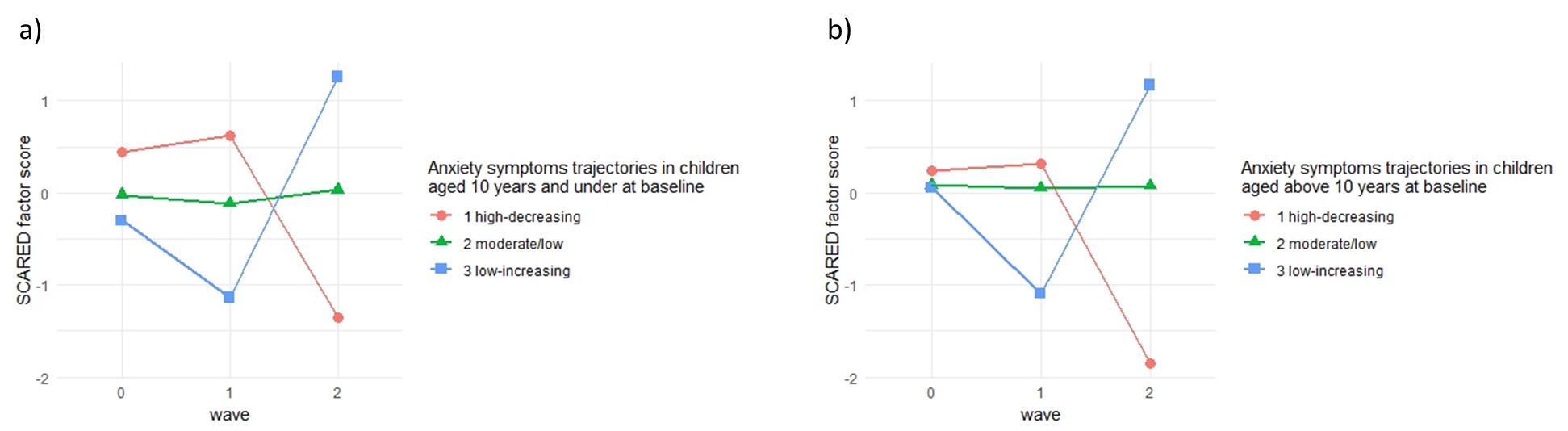
**

**Figure S3. Anxiety symptoms trajectories: a) children aged 10 years and under at baseline, and b) children aged above 10 years at baseline**

**Table S1. SCARED questionnaire measurement invariance across time**

| **Model and comparisons** | **Fit Statistics** | |  |  |  |  |  |  |  |
| --- | --- | --- | --- | --- | --- | --- | --- | --- | --- |
|  | **Χ² (df)** | **ΔΧ²** | **p-value** | **CFI** | **ΔCFI** | **SRMR** | **ΔSRMR** | **RMSEA** | **ΔRMSEA** |
| **M1: Configural** | 37.23 (2,337) |  |  | 1.00 |  | 0.044 |  | 0.002 |  |
| **M2: Metric** | 86.05 (2,417) |  |  | 1.00 |  | 0.063 |  | 0.001 |  |
| **M3: Scalar** | 119.80 (2,497) |  |  | 1.00 |  | 0.063 |  | 0.002 |  |
| **M4: Strict** | 128.52 (2,579) |  |  | 1.00 |  | 0.063 |  | 0.002 |  |
| **M2 − M1** |  | 78.41 | 0.529 |  | 0 |  | 0.019 |  | -0.001 |
| **M3 − M2** |  | 197.16 | 0.000 |  | 0 |  | 0 |  | 0.002 |
| **M4 − M3** |  | 75.92 | 0.671 |  | 0 |  | 0 |  | 0 |

**Table S2. SCARED questionnaire questions and loadings**

| **Item** | **Question** | **Factor Loading** |
| --- | --- | --- |
| 1 | When I feel frightened, it is hard for me to breathe | 0.568 |
| 2 | I get headaches when I am at school | 0.461 |
| 3 | I don’t like to be with people I don’t know well | 0.329 |
| 4 | I get scared if I sleep away from home | 0.486 |
| 5 | I worry about other people liking me | 0.519 |
| 6 | When I get frightened, I feel like passing out | 0.693 |
| 7 | I am nervous | 0.510 |
| 8 | I follow my mother or father wherever they go | 0.226 |
| 9 | People tell me that I look nervous | 0.539 |
| 10 | I feel nervous with people I don’t know well | 0.536 |
| 11 | My I get stomachaches at school | 0.471 |
| 12 | When I get frightened, I feel like I am going crazy | 0.746 |
| 13 | I worry about sleeping alone | 0.563 |
| 14 | I worry about being as good as other kids | 0.483 |
| 15 | When I get frightened, I feel like things are not real | 0.618 |
| 16 | I have nightmares about something bad happening to my parents | 0.618 |
| 17 | I worry about going to school | 0.680 |
| 18 | When I get frightened, my heart beats fast | 0.629 |
| 19 | I get shaky | 0.673 |
| 20 | I have nightmares about something bad happening to me | 0.628 |
| 21 | I worry about things working out for me | 0.501 |
| 22 | When I get frightened, I sweat a lot | 0.546 |
| 23 | I am a worrier | 0.708 |
| 24 | I get really frightened for no reason at all | 0.738 |
| 25 | I am afraid to be alone in the house | 0.496 |
| 26 | It is hard for me to talk with people I don’t know well | 0.453 |
| 27 | When I get frightened, I feel like I am choking | 0.819 |
| 28 | People tell me that I worry too much | 0.636 |
| 29 | I don’t like to be away from my family | 0.540 |
| 30 | I am afraid of having anxiety (or panic) attacks | 0.680 |
| 31 | I worry that something bad might happen to my parents | 0.509 |
| 32 | I feel shy with people I don’t know well | 0.490 |
| 33 | I worry about what is going to happen in the future | 0.480 |
| 34 | When I get frightened, I feel like throwing up | 0.684 |
| 35 | I worry about how well I do things | 0.403 |
| 36 | I am scared to go to school | 0.748 |
| 37 | I worry about things that have already happened | 0.582 |
| 38 | When I get frightened, I feel dizzy | 0.802 |
| 39 | I feel nervous when I am with other children or adults and I have to do something while they watch me (for example: read aloud, speak, play a game, play a sport) | 0.505 |
| 40 | I feel nervous when I am going to parties, dances, or any place where there will be people that I don’t know well | 0.533 |
| 41 | I am shy | 0.305 |

**Table S3. Intercepts and slopes of the best Growth Mixture Model**

|  | **coef** | **Se** | **Wald** | **p-value** |
| --- | --- | --- | --- | --- |
| intercept class1 | 0.38833 | 0.10379 | 3.741 | 0.00018 |
| intercept class2 | 0.02914 | 0.02503 | 1.164 | 0.24435 |
| intercept class3 | -0.19168 | 0.16507 | -1.161 | 0.24555 |
| Slope time1 class1 | 0.11020 | 0.10111 | 1.090 | 0.27577 |
| Slope time1 class2 | -0.06250 | 0.02535 | -2.465 | 0.01370 |
| Slope time1 class3 | -0.85034 | 0.16421 | -5.178 | 0.00000 |
| Slope time2 class1 | -1.76858 | 0.11792 | -14.998 | 0.00000 |
| Slope time2 class2 | 0.02946 | 0.03003 | 0.981 | 0.32671 |
| Slope time2 class3 | 1.27338 | 0.20970 | 6.072 | 0.00000 |
